# Carney complex- why thorough medical history taking is so important - report of three cases and review of the literature

**DOI:** 10.1007/s12020-022-03209-2

**Published:** 2022-10-18

**Authors:** B. Harbeck, J. Flitsch, I. Kreitschmann-Andermahr

**Affiliations:** 1 III. Department of Medicine, University Medical Center Hamburg-Eppendorf, Hamburg, Germany; 2 MVZ Amedes Experts, Endocrinology, Hamburg, Germany; 3 Department of Neurosurgery, University Medical Center Hamburg-Eppendorf, Hamburg, Germany; 4 Department of Neurosurgery and Spine Surgery, University Hospital Essen, Essen, Germany

**Keywords:** Myxoma, Primary pigmented nodular adrenocortical disease, Carney complex, Patient history, Surveillance

## Abstract

**Purpose:**

To present a new case series and to review the literature on Carney complex (CNC) with an emphasis on highlighting key clinical features of the disease and pointing out possibilities of shortening the diagnostic process.

**Method:**

Searches of PubMed, identifying relevant reports up to April 2022.

**Results:**

CNC is a rare, autosomally dominant inherited neoplasia -endocrinopathy syndrome with high clinical variability, even among members of the same family. Data on length of diagnostic process are scarce with numerous case series reporting a diagnostic delay of decades. Suggestions to shorten the diagnostic process includes awareness of the multi-faceted clinical presentations of CNC, thorough history taking of index patients and family members and awareness of diagnostic pitfalls. Importantly, unusual symptom combinations should alert the clinician to suspect a rare endocrinopathy syndrome such as CNC. Already present and coming on the horizon are databases and novel phenotyping technologies that will aid endocrinologists in their quest for timely diagnosis.

**Conclusion:**

In this review, we examine the current state of knowledge in CNC and suggest avenues for shortening the diagnostic journey for the afflicted patients.

## Introduction

Carney complex (CNC) is a very rare multiple neoplasia syndrome with an autosomal dominant inheritance (70%) or can occur sporadically (30%) as a de novo genetic variant [1]. It was discovered by Dr. Adrian J. Carney in 1985 as “the complex of myxomas, spotty pigmentation and endocrine overactivity” [2] and named after him „Carney´s complex” one year later [3]. CNC is rare and intricate, with clinical features varying greatly from one person to another, even among members of the same family. Although many of the signs and symptoms of CNC become apparent during adolescence or early adulthood, the correct diagnosis is oftentimes delayed, sometimes by decades [4–6].

Starting with the description of three new cases, this review aims to highlight essential diagnostic and therapeutic features of CNC, taking into account the literature on this disorder with regard to prevalence, etiology, pathophysiological mechanisms and treatment. To this end, we searched PubMed for any combination of the term “Carney complex“ with catchphrases relating to diagnosis, signs, symptoms, diagnostic delay, etc. All articles reporting original data and review articles on CNC in peer-reviewed journals were screened for inclusion in the present review. We also searched the reference lists of articles identified by this search strategy. Finally, we screened for published case reports on CNC. In particular, we included the papers with the highest clinical relevance highlighting the diagnostic process and symptoms and severity of the afflicted patients.

## Case series

### Patient 1

A 29 -year-old female suffering from previously undiagnosed Cushing´s syndrome (CS) for one year with weight gain, arterial hypertension and a moon face was admitted to the first author’s endocrine department. Laboratory tests in an external hospital had revealed adrenocorticotrophic (ACTH) hormone independent hypercortisolemia with lack of cortisol suppression in the 8 mg dexamethasone suppression test and missing stimulation of cortisol in the corticotropin releasing hormone (CRH) test. Magnetic resonance imaging (MRI) of the adrenal glands was unremarkable. The patient underwent bilateral adrenalectomy and histological work-up of the tissue was consistent with primary pigmented nodular adrenocortical disease (PPNAD). Postoperatively, she was put on hydrocortisone and fludrocortisone replacement. Her medical history revealed recurrent excisions of myxolipomas since the age of 14. Meantime, laboratory data showed an increase of prolactin and insulin-like growth factor I (IGF-I; see Table 1). Growth hormone (GH) was not suppressed in oral glucose tolerance testing (oGTT), indicating acromegaly. Pituitary MRI demonstrated a 5 mm microadenoma which was also successfully removed and histologically classified as a densely granulated somatotroph adenoma with prolactin co-expression (1%). During medical work-up, she reported that her younger sister suffered from similar symptoms.

**Table 1:** Important laboratory data of patients 1 and 2 at initial presentation

	Patient 1	Patient 2
ACTH (7–63 ng/l)	**1.91**	**1.29**
Cortisol (133–537 nmol/l)	371.0	**1696.0**
Urinary cortisol (3.0–43.0 mcg/d)	**328.3**	**2487.0**
23 h Salivary cortisol (<4,1 ng/ml)	**18.4; 14.4**	**25.2**
Cortisol following 1 mg dexamethasone suppression test (mcg/l)	**145.0**	**>634.0**
Cortisol following 8 mg dexamethasone suppression test (nmol/l)	**264.0**	Refused by patient
CRH-test	Cortisol: **249.0** (0 min) -**255.0** (15 min) -**251.0** (30 min) -**250.0** (45 min) -**244.0** (60 min) -**244.0** (90 min) - **242.0** (120 min)	Cortisol: **1102.0** (0 min) -**1185.6** (15 min) -**1145.1** (30 min) - **1108.6** (45 min) -**1041.6** (60 min) -**1038.2** (90 min) -**1038.0** min (120 min)
	ACTH: < **1,5** (0 min) -**1.62** (15 min) -**1.77** (30 min) - **2.09** (45 min) -**1.95** (60 min) -**1.78** (90 min) -**1.69** (120 min)	ACTH: **1.8** (0 min) - **2.0** (15 min) - **3.8** (30 min) - **3.0** (45 min) - **2.9** (60 min) -**2.8** (90 min) -**2.68** (120 min)
Prolactin (4.8–23.3 mcg/l)	**74.7**	21.2, **101.5** (12 weeks later)
GH (0–6.88 mcg/l)	3.27	
IGF-1 (117–329 ng/ml)	**349.0** **464.8** (4 weeks later)	218.0, **958.2** (12 weeks later)
GH suppression test	**2.05** (0 min) - **1.19** (30 min) - **1.49** (60 min) - **1.56** (120 min)	
oGTT (mg/dl)	70 (0 min) - 190 (60 min) -116 (120 min)	**103** (0 min) - 229 (60 min) - **256** (120 min)
TSH (0.27–4.2 mIU/ml)	1.66	1.14
fT3 (2.0–4.4 pg/ml)	3.27	**1.3**
fT4 (9.3–17.0 ng/l)	10.48	**8.1**
FSH (mIE/ml)	7.4	**<0.1**
LH (mIE/ml)	18.6	**<0.1**
Estradiol (pg/ml)	36.2	**<5.0**

*TSH* thyroid stimulating hormone, *FSH* follicle-stimulating hormone, *LH* luteinizing hormone

Pathological findings are typed in bold

### Patient 2

Pushed by her older sister, a 20-year-old female admitted to the first author’s endocrine department. She also showed a moon face, suffered from tiredness, violaceous striae on her arms, arterial hypertension and diabetes mellitus. Small skin tumors had been removed in recent years compatible with myxolipomas. Serum cortisol and urinary cortisol excretion were clearly elevated. ACTH was in the lower range (Table 1). Suspecting CS, a 1 mg dexamethasone suppression test was performed that did not show cortisol suppression and a CRH test that did not induce a cortisol increase. MRI of the adrenal glands was compatible with bilateral PPNAD, which was confirmed after adrenalectomy. After surgery, hydrocortisone and fludrocortisone replacement were commenced. Since laboratory results were indicative of secondary hypothyroidism and revealed elevated IGF-I (see Table 1) and prolactin (see Table 1) levels, an MRI of the pituitary was done, showing an invasive pituitary macroadenoma, which was surgically treated and classified as somatomammotroph adenoma. At follow-up visits, the patient regained her normal face shape, blood pressure and blood glucose normalized.

### Patient 3

In the course of her daughters´ treatment, patient 3 was seen in the first author’s endocrine department in 2019. In 1987, at the age of 23 years she had undergone left-sided adrenalectomy due to CS. According to her operation report “multinodular adenomatous hyperplasia“ was found and interpreted back then as a sign of “ACTH- dependent Cushing’s disease“. Nevertheless, no further evaluation of the pituitary gland was performed. Two years later the patient developed hypercortisolism again and the right adrenal gland was also subtotally removed. After the retirement of her old endocrinologist, she was treated with hydrocortisone, fludrocortisone and dehydroepiandrosterone (DHEA) replacement by her new endocrinologist due to suspected Addison’s disease. Due to headache, MRI of the pituitary was performed in 2001, revealing a pituitary microadenoma, which according to laboratory results was hormonally inactive. In addition, a fibroadenoma of the breast was removed, and further ones were detected in the following years. 2010, at the age of 46 years, the patient was diagnosed with atrial myxoma, successfully treated by cardiac surgery.

### Genetics

Genetic testing was performed in the three patients, detecting a variant of the *PRKAR1A* gene, which has not been described in literature so far (variant c.156delA p.(Glu52Aspfs*77), heterozygous). In accordance with the patients’ clinical findings, imaging manifestations, and gene variants, the diagnosis of CNC was made.

### Follow-up

Following the diagnosis CNC, lifelong medical aftercare was initiated in all patients. Apart from fibroadenomas of the breasts and thyroid cysts in patients 1 and 2, follow-up visits of the two daughters were uneventful. However, in patient 3, a recurrent big left atrial myoma (2,8 × 2,2 cm) was uncovered. The myxoma was resected with the reconstruction of the atrial septal septum.

### Carney complex

#### Epidemiology

The prevalence of CNC is unknown [4, 7]. Published reports indicate more than 750 affected individuals worldwide [1, 8, 9]. However, patients diagnosed in the past with recurrent atrial myxomas or lentigines might be reclassified today as CNC. Males and females are affected to an equal extent. Clinical features of the disorder may be present at birth with a median age of diagnosis reported to be around 20 years of age [1, 9]. People with a sporadic mutation typically show mild symptoms later in life.

#### Clinical features and diagnostic criteria

Table 2 gives an overview about the main clinical features and symptoms that are “warning signs” pointing to CNC. Due to the broad spectrum of symptoms and the enormous variability of clinical presentations, it is essential to obtain a thorough medical history. Our case series underlines this compelling necessity.

**Table 2:** Clinical features and warning signs of CNC

Manifestations	Frequency	Symptoms	Age at manifestation	Complications	CAVEAT:
Skin lesions	more than 80%, most common [34]	lentigines (70–80%), blue nevus (40%), cutaenous myxomas (30–50%) [34]	adolescence		among the earliest and most easy to detect [34]
Cardiac tumors	20–40% [8]	cardiac myxomas	mean age: females 28.7 years, males 25.0 years [35]	intracardiac obstruction and embolization [36], recurrences in 44% [35]	leading cause of death [1, 37]
Pituitary tumors	up to 80%: increased levels of GH, IGF-I or prolactin, pathological GH response to oGTT; incidence of acromegaly in this group due to pituitary adenoma 10–12% [38–40]; some cases of prolactinomas [8]	rarely signs of acromegaly	20–30 years	complications of untreated acromegaly	histological work-up of the tumor resections: frequently immunostaining not only for GH but also for prolactin and TSH alpha subunit [7]
Adrenocortical tumors	25-60% PPNAD, f>m [10, 41]	hypercortisolism due to PPNAD, subclinical or overt (1/4) [42]	second and third decades [1]	very few cases of adrenocortical carcinomas [43]	most common endocrine manifestation
Ophthalmologic manifestations	up to 70% [44]	eyelid myxomas, facial and palpebral lentigines and pigmented lesions of the caruncle or conjunctival semilunar fold [44, 45]	whole lifespan		usually precede more serious manifestations, e.g., cardiac myxoma [44]
Psammomatous melanotic schwannomas	up to 10%	most often found in the gastrointestinal tract and in the paraspinal sympathetic chain (28%; [7, 46]; pain and radiculopathy	30–40 years [47]	malignancy rate: about 10% [47, 48]	
Thyroid neoplasms	2/3 thyroid nodules (cysts (75%, [7]) or adenomas (25%, [49])		whole lifespan but often during the first 10 years of life [41, 50].	malignancy rate up to 10% (follicular, papillar cancer) [51, 52]	
Breast tumors		breast myxomas, myxoid fibroadenomas and ductal adenomas [53, 54].	after puberty [53, 54]		typically, multiple
Ovarian lesions	common [55, 56]	ovarian cysts, serous cystadenomas and cystic teratomas	after puberty	rarely progress to ovarian carcinoma [55, 56]	
Testicular tumors	up to 75%	large cell-calcifying Sertoli cell tumors (LCCSCT) [57, 58]	childhood	increased risk of reduced fertility [59, 60] and malignancy in adults [61, 62], malignancy rate 17% [63]	physicians should be aware of these very rare testicular tumors representing <1% of all testicular neoplasms [57, 58] and their possible connection to CNC
Bone lesions	1% osteochondromyxoma [7], 31.6% vertebral nodular lesions [64]		osteochondromyxoma: usually early childhood [7]; vertebral lesions interpreted as osteochondromyxomas 30-40 years [64]	osteochondromyxomas have the potential to be locally invasive and to recur [65]	typically associated with CNC [66]
Other tumors		pancreatic neoplasias [67] as well as hepatic [68], colonic [69] or gastric tumors [70]			

**Fig. 1:**
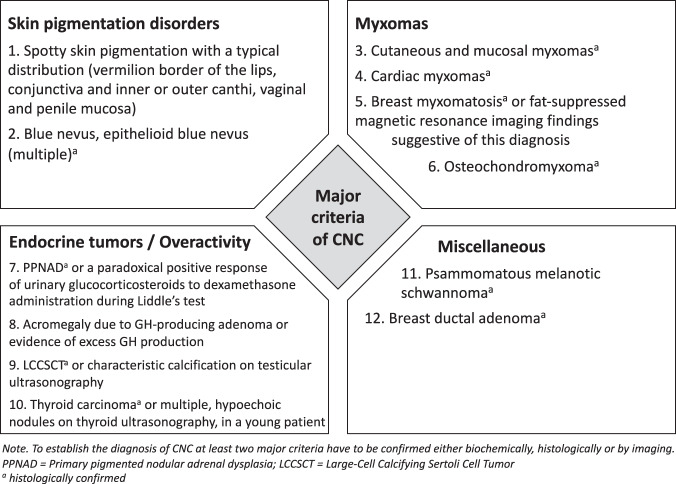
Major Criteria of Carney Complex (CNC)

According to the revised diagnostic criteria published in 2001, patients must either: 1) exhibit two of the manifestations of the disease listed in Fig. 1, or 2) exhibit one of these manifestations and meet one of the supplemental criteria (an affected first-degree relative or an inactivating mutation of the PRKAR1A gene; [1]). By this approach, the sensitivity of diagnosis is nearly 98%.

#### Genetics

CNC is inherited in an autosomal dominant manner or occurs sporadically in a de novo gene mutation [1]. The “Carney Complex gene“ *PRKAR1A* was found by Dr Constantine Stratakis in March 2000. More than two third of CNC patients harbor inactivating variants of the regulatory subunit type 1A of this cAMP-dependent protein kinase gene (on chromosome17q22-24; CNC 1 locus; [10, 11]). Also, 80% of patients with PPNAD show a variant in the *PRKAR1A* gene [10, 12]. More than 130 different *PRKAR1A* variants in over 400 unrelated families have been identified, most of them in single families only [7]. Additionally, a second genetic cause on the short arm of chromosome 2 is being discussed (2p16; CNC 2 locus; [13–15]). In 2014, a copy number gain of the short arm of chromosome 1 including the *PRKACB* gene was found in a single person with CNC [16] Additionally, inactivating variants of *PDE11A* have been described in patients with CNC [15]. Other disease-related genes include activating *PRKACA* variants [17]. The genes involved in CNC encode the essential players of the cAMP/PKA pathway leading in case of inactivity to an increase of cAMP signaling as well as copy-number gains of genes encoding the catalytic subunits as a sign of dysregulation of the cAMP/PKA pathway [15].

#### Treatment

Due to the complexity of this syndrome each specific manifestation should be addressed separately. A causal therapy is not established. Surgical treatment of CNC-associated tumors is the treatment of choice in most cases. Patients should be referred to an endocrine center with treatment in a multi-disciplinary team. Genetic counselling should be offered to all patients with known CNC, patients with CNC diagnostic criteria but without any family history of CNC as well as first–degree relatives. Psychological support should be included to enable affected individuals to process the impact of the disease and its possible consequences for their life and family planning.

#### Prognosis

The risk of adrenocortical carcinoma, thyroid, colorectal, liver and pancreatic cancers is increased in CNC patients [18]. Moreover, ovarian cancers in women and tumors of the testicles in men involving the Sertoli or Leydig cells have been reported [18]. However, with careful surveillance, life expectancy can be normal. Most patients die due to complications of heart myxomas (e.g., cardiac arrhythmias or emboli), cancer or metastatic PMS [19–21].

#### Surveillance

Careful surveillance is essential in patients diagnosed with CNC. Patients should be seen by a specialist at least once a year. Table 3 shows the suggested follow-up intervals [22, 23].

**Table 3:** Surveillance program

	Children [1, 7]	Adolescents and adults [7, 23]
*Annually*	Echocardiogram (biannually if a cardiac myxoma has already been excised); testicular ultrasound for boys; monitoring of growth rate and pubertal stage	Echocardiogram (biannually if a cardiac myxoma has already been excised); testicular ultrasound; skin evaluations; endocrine tests: serum: GH, IGF-I, prolactin, urine: free cortisol and other testing for Cushing´s syndrome if appropriate
*Baseline and/or if required*		Thyroid ultrasound; transabdominal ultrasound of the ovaries; imaging as required (e.g., CT of the adrenals, MRI spine/chest/abdomen/retroperitoneum/pelvis); oGTT if needed

## Discussion

Our case series illustrates what endocrinologists know to be the sobering truth not only in CNC but also other patients with rare endocrine disorders: a protracted journey to diagnosis, ranging from 15 years in patient No. 1 to 32 years in patient No. 3, even though key features of the disease were present much earlier. Such a long time to diagnosis is also reported in numerous case presentations of CNC [4–6, 24, 25]. Interestingly, the meaning of a detailed enquiry of the index patient and other family members is not discussed in most reports. In addition, affected individuals are typically seen by many different specialists who, in the face of high caseloads and time pressures, may just focus on their own area of expertise without noticing other relevant diagnostic clues. Also, patients have preconceived notions of medical disciplines and their competence for individual aspects of disease presentation [26, 27]. This, on the patient side, can lead to monosymptomatic symptom presentations without forwarding further important medical information on their own accord.

Improving the lag in time to diagnosis in combination with regular follow-ups is vital in CNC to improve patients’ outcome as our patient series illustrates. Although only a fraction of endocrinologists will ever encounter patients with this syndrome, the first and most important step to diagnosis is the knowledge of the multifaceted clinical manifestations of CNC, enabling a detailed enquiry into the patient’s and family history.

It also makes sense for the clinician to be aware of Bayes’ theorem, an 18th century mathematical rule, oftentimes used in modern evidence-based medicine. The mathematical equation relates current to previous probabilities, taking into account new pieces of evidence [28, 29] and elucidates how the initially very low previous probability of a true diagnosis of a rare disease is influenced and improved when a new symptom or test is added to the diagnostic process [29, 30]. What does that imply for the diagnosis of CNC? As in other rare endocrine diseases, unusual symptom combinations such as early onset osteoporosis in combination with rapid weight gain and proximal muscle weakness in Cushing’s disease [27] should put physicians on the lookout to suspect a rare endocrine syndrome. In CNC, the co-occurrence of multiple myxomas and endocrine disorders or the presence of cardiac myxomas and typical skin lesions are clear indications for screening [31]. Additionally, PPNAD, as a rare primary bilateral adrenal defect, should alert treating physicians to look for CNC in patients with this disorder. In a series of 88 patients with PPNAD, 40 cases were part of CNC [32]. Endocrinologists should also know that modern databases and next-generation phenotyping technologies, such as software that can automatically detect characteristic syndromic facial features from a portrait photograph [33], and other machine learning technologies, become increasingly helpful in detecting rare diseases. Nevertheless, thorough medical history taking and awareness of unusual symptom combinations will remain important cornerstones of uncovering rare diseases for years to come.
